# A Study on the Formation of Flavor Substances by Bacterial Diversity in the Fermentation Process of Canned Bamboo Shoots in Clear Water

**DOI:** 10.3390/foods12183478

**Published:** 2023-09-19

**Authors:** Ke Li, Ning Gao, Jiaojiao Tang, Huiqin Ma, Jiayan Jiang, Yufan Duan, Zongjun Li

**Affiliations:** 1 College of Food Science and Technology, Hunan Agricultural University, Changsha 410128, China; likeqin1141@163.com (K.L.); mygaoning1213@163.com (N.G.); tangjiao@stu.hunau.edu.cn (J.T.); mbomb0730@163.com (H.M.); jjyxmg@stu.hunau.edu.cn (J.J.); 15848169722@163.com (Y.D.); 2 Hunan Province Key Laboratory of Food Science and Biotechnology, Changsha 410128, China

**Keywords:** bamboo shoots, fermentation process, *Lactococcus lactis* TJJ2, tyrosine

## Abstract

Canned bamboo shoots in clear water could produce a unique flavor through bacterial diversity via the fermentation process. *Weissella*, *Streptococcus*, *Leuconostoc*, *Acinetobacter*, *Lactococcus* and *Lactobacillus* were the main microorganisms. Tyrosine was the most abundant free amino acid (FAA), which had a negative correlation with *Lactococcus*. Ten kinds of flavor substances, such as 3-methyl-1-butanol, acetic acid, 2-phenylethyl ester, benzene acetaldehyde, benzoic acid and ethyl ester, were important influential factors in the flavor of fermented bamboo shoots. Through the verification test of tyrosine and phenylalanine decarboxylase, it was found that *Lactococcus lactis* TJJ2 could decompose tyrosine and phenylalanine to produce benzaldehyde and benzene acetaldehyde, which provided the fermented bamboo shoots with a grassy aroma.

## 1. Introduction

Fermentation is an important traditional method of vegetable preservation. In many modern industrialized food processing processes, the principles and technology of traditional fermentation are still combined to obtain the food characteristics that people need so that fermented vegetables form new flavor, texture, nutrition and storage processing characteristics, such as common pickles [1], sauerkraut [2] and so on. The fermentation process of canned bamboo shoots in clear water is a process in which nutrients, microbial diversity and flavor undergo significant changes, including microbial enzyme catalysis, amino acid metabolism, esterification reaction, etc. These reactions are affected by raw materials, microbial species, fermentation time, pH and temperature. Fermentation improves the nutritional value of bamboo shoots, for example, by causing changes in crude fiber content, protein, phenols, flavonoids, tannin and amino acid composition.

Darmayanti et al. [3] reported that a reduction in HCN content was found in fermented kimchi of Taba bamboo shoots (Gigantochloa nigrociliata (Buese) Kurz). Sarangthem and Singh [4] also reported that fermentation reduces the level of cyanogen in bamboo shoots. Another recent study [5] reported that cyanogenic glycosides in traditionally prepared bamboo shoot products (e.g., hirring, soibum, soidon, hecche, ekung and eup) were within the limit (<10 ppm).

Devi and Singh [6] showed that the soluble protein content in fermented buds increased from 3.1% to 7.8% and 8.1% on the third and fifth day of fermentation, respectively. In another study by Agrahar-Murugkar and Subbulakshmi [7], fermented bamboo lungs were found to have better nutritional value (8.5%) in terms of protein content.

A large proportion of the flavor compounds in fermented foods are derived from carbohydrate degradation, protein decomposition, and amino acid and fatty acid metabolism under the action of microorganisms. Due to the high protein content in bamboo shoots, FAAs have become important flavor precursors [8]. Alcohols, aldehydes, carboxylic acids and esters are mainly produced through amino acid metabolic pathways such as transamination and decarboxylation [9].

Fermented bamboo shoots have a variety of microorganisms. *Lactobacillus brevis*, *Lactobacillus coryniformis* and *Lactococcus lactis* can enrich the flavor, aroma and acidity of fermented foods [10,11]. Hata et al. isolated a strain of *Enterococcus faecalis* from fermented bamboo shoots and demonstrated that this strain could produce bacteriocins and inhibit the growth of pathogenic bacteria [12]. The detection of microorganisms in bamboo shoots (*Dendrocalamus latiflorus*) during the pickling process showed that *L. lactis* and *Weissella* successively became the dominant bacteria, the total acid content increased and the pH values decreased.

The microbial changes in the fermentation process of canned bamboo shoots in clear water are mainly focused on bacterial diversity. In-depth knowledge of the changes in bacterial diversity and its correlation with flavor substances is essential to better understand the contribution of bacteria and to develop specific starter cultures suitable for bamboo shoot fermentation. This study investigated the bacterial diversity of naturally fermented bamboo shoots in a factory environment, and the relationship between flavor compounds and lactic acid bacteria (LAB) was revealed through computational approaches and validation tests. It provided a theoretical basis and guidance for standardizing the control of the fermentation process, ensuring the quality stability of canned bamboo shoots in clear water, shortening the fermentation time and improving production efficiency.

## 2. Materials and Methods

### 2.1. Preparation Process of Samples

The preparation process and volatile flavor compound (VFC) detection were the same as that of [13]. The bamboo shoots were fermented for 0 h (FBSA), 36 h (FBSB) and 72 h (FBSC). All fermented bamboo shoot samples were collected at Hunan Jingshi Agricultural Science and Technology Co., Ltd. (Taojiang, China), stored immediately at −80 °C and transported to the laboratory for further analysis.

### 2.2. Analysis of Microbial Diversity

#### 2.2.1. DNA Purification and PCR Amplification

DNA from the samples was extracted using the cetyltrimethylammonium bromide (CTAB) method [14]. Briefly, the samples were centrifuged at 4000× *g* for 10 min, the supernatant was discarded and the pellets were resuspended in 5 mL of CTAB lysis buffer. Cell lysis was achieved by incubating the samples at 65 °C for 60 min. DNA was then extracted twice using an equal volume (5 mL) of chloroform: isoamyl alcohol (24:1; Sigma–Aldrich, St. Louis, MO, USA) each time. Cellular fractions were separated by centrifuging the samples at 8000× *g* for 15 min and the process was repeated. DNA was precipitated at −20 °C overnight in 5 mL of isopropanol: 7.5 M ammonium acetate (9:1; Sigma–Aldrich). DNA was harvested by centrifugation at 8000× *g* for 15 min. Finally, DNA pellets were washed twice in 5 mL of 70% (*v*/*v*) ethanol (Sigma–Aldrich) and samples were collected by centrifugation at 8000× *g* for 10 min. Then, DNA was diluted with sterile water to 1 ng/µL and used as a template. The V3-V4 region of the bacterial 16S rRNA gene was amplified with primers 515F (5′-GTGCCAGCMGCCGCGGTAA-3′) and 806R (5′-GGACTACHVGGGTWTCTAAT-3′). PCR amplification was performed using specific primers with barcode and high-fidelity enzymes. According to the concentration of PCR products, the samples were mixed equally, and then the PCR products were detected by 2% agarose gel and further purified using the AxyPrep DNA Gel Extraction Kit (Axygen Biosciences, Union City, CA, USA) and quantified using QuantiFluor™-ST (Promega, Madison, WI, USA) according to the manufacturer’s protocol. Purified amplicons were pooled in equimolar amounts and paired-end sequenced (2 × 300) on an Illumina MiSeq platform (Illumina, San Diego, CA, USA) according to the standard protocols by Majorbio Bio-Pharm Technology Co., Ltd. (Shanghai, China).

#### 2.2.2. High-Throughput Sequencing and Sequence Analysis

The target bands were recovered from the gel recovery kits, and a library was constructed with the TruSeq^®^ DNA PCR-Free Sample Preparation Kit and quantified by Qubit and Q-PCR. After that, NovaSeq6000 was used for computer sequencing. Raw fastq files were demultiplexed, quality filtered by Trimmomatic and merged by FLASH using the criteria described in [15]. The trimmed and unique sequences were used to define the number of operational taxonomic units (OTUs) at the 97% similarity level. The α-diversity was estimated by the Chao1 richness and Shannon diversity indices using Mothur software V1.45.1 [16]. Beijing Nuohe Zhiyuan Technology Co., Ltd. (Beijing, China) completed the microbial diversity test of this study.

### 2.3. Determination of FAAs

The samples (0.5 g) were mixed with 5 mL of 50% ethanol (containing 0.01 mol/L HCl), followed by ultrasonic extraction at 40 °C for 30 min. The solution was centrifuged at 10,000× *g* for 5 min at 25 °C, and 1 mL of the supernatant was aspirated and lyophilized. The dried extract was dissolved in deionized water, filtered through a 0.22 µm membrane and tested by an amino acid automatic analyzer (S-433D, Sykam Inc., Eresing, Germany).

### 2.4. Isolation, Purification and Identification of L. lactis TJJ2

Sterile sampling of 25 g bamboo shoots was diluted to an appropriate gradient using 0.9% sterile normal saline. The 200 μL sample was absorbed into the MRS solid medium for coating and incubated at 37 °C until colonies formed. Several different types of single colonies were selected and inoculated in plates by the cross method and placed in an incubator at 37 °C for static culture. This operation was repeated 3~5 times until a single colony with consistent morphology grew. The isolated strains were stored in 40% (volume fraction) glycerol at −80 °C for further use.

The isolated and purified strains were preliminarily identified by colony morphological observation, Gram staining, physiology and molecular biology. The colonies were collected from the solid medium using a cotton swab to prepare a bacterial suspension with a turbidity equivalent to 2 McFarland. The bacterial solution was placed in a 50 CHL medium ampoule (Beijing ZhijieFangyuan Technology Co., Ltd., Beijing, China) and covered with paraffin oil. The culture was incubated at 37 °C for 48 h and the color change was observed.

The screened strains were inoculated in the MRS liquid medium and cultured at 37 °C for 24 h. The genome of the target strain was obtained according to the requirements of the bacterial genome rapid extraction kit (Hangzhou Beiwo Medical Technology Co., Ltd., Hangzhou, China). The primer sequences were 27F (5′-AGAGTTTGATCCTGGCTCAG-3′) and 1492R (5′-TACGACTTAACCCCAATCGC-3′). The target fragment was 1540 bp. The PCR conditions were as follows: 96 °C pre-denaturation for 5 min, denaturation for 30 s, 68 °C annealing for 30 s, 72 °C extension for 1.5 h and 35 cycles, and finally extension at 72 °C for 10 min. The PCR product was stored at 4 °C and then sent to Sangon Bioengineering Co., Ltd. (Shanghai, China) for 16S rRNA sequencing. The spliced results were entered into NCBI (www.ncbi.nlm.nih.gov accessed on 12 March 2023) for BLAST alignment to find the strain with the highest homology to the target gene sequence and its sequence. The phylogenetic tree was constructed by MEGA 7.0 software.

### 2.5. Detection of Decarboxylase by Cultivation Method

The decarboxylation medium was prepared according to with a modification: the broth contained tyrosine at a concentration of 1% (*w/v*) and a pH indicator (bromcresol purple). *L. lactis* TJJ2 isolated from the fermented bamboo shoots was inoculated in thedecarboxylase medium and cultured at 37 ℃ for 48 h, and 5 mL samples were taken for VFCs.

### 2.6. Statistical Analysis

The differences among treatments were analyzed by a one-way analysis of variance (ANOVA) with multiple comparison Tukey tests using IBM SPSS 17.0 (SPSS Inc., Chicago, IL, USA) and the significance level in the analyses was considered at *p* < 0.05. The relationship between bacterial genera and FAAs was assessed by Spearman’s correlation coefficient at |ρ| > 0.8, with statistical significance (*p* < 0.01), and visualized via TBtools (version 1.068) [17,18]. Canonical correlation analysis (CCA) was used to assess the relationship between bacterial genera and characteristic VFCs in Canoco (version 5.0). All experiments were performed in triplicate and the data are expressed as the mean ± standard deviation.

## 3. Results and Discussion

### 3.1. Changes in Bacterial Diversity during Fermentation

**Figure 1:**
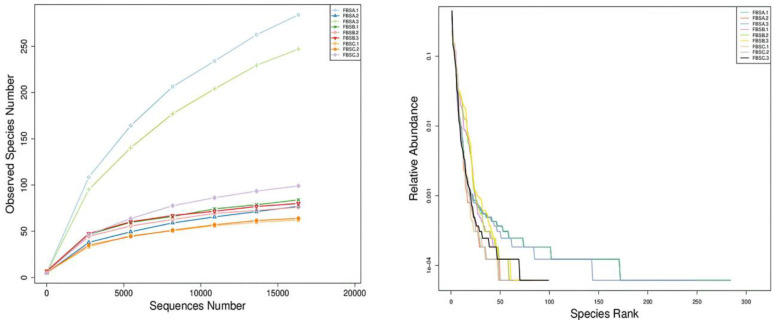

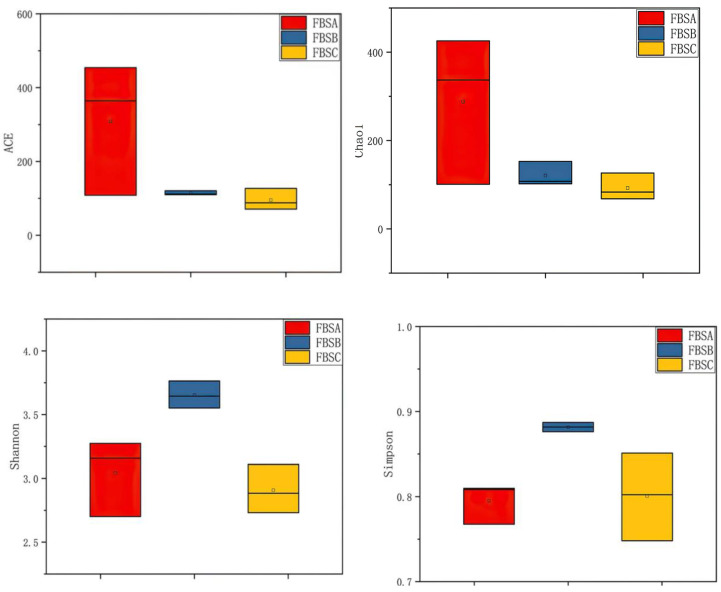
Rarefaction curve, rank abundance curve and alpha diversity index. Supplementary: Figure 1 shows the changes of microorganisms in bamboo shoot juice during the fermentation of bamboo shoots, where the top two graphs represent the dilution index curves, the middle two represent the Index of rarefaction curve, and the bottom two graphs represent the α-diversity; and the six graphs represent the diversity indices during 72 h of fermentation.

The diversity index reflects species abundance and uniformity. As shown in Figure 1, the rarefaction curve of the three fermentation periods was gentle, indicating that the sequencing data were sufficient to cover the diversity of bacterial species in the samples. In the rank abundance curve, the shapes were similar and tended to be flat, indicating that the species composition of bamboo shoot bacteria in the three fermentation periods was similar. The ACE and Chao1 indices reflected species richness in the samples and FBSA had the highest abundance of bacteria during the first fermentation period. The Shannon and Simpson indices indicated species diversity in a sample and FBSB was the highest among all the studied samples. The species gradually became uniform in the early fermentation stage and the variety of bacteria increased during fermentation.

### 3.2. Cluster Analysis of Bacterial

**Figure 2:**
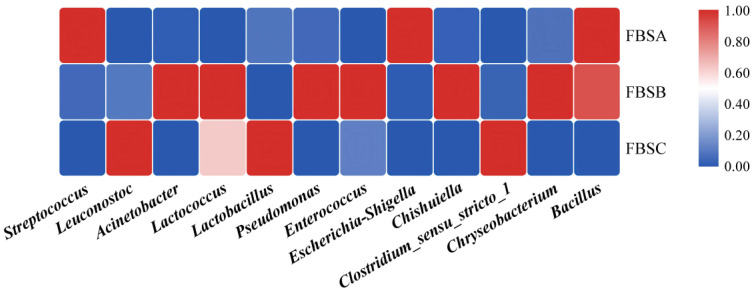
Heatmap of bacterial diversity at the genus level. Supplementary: where the horizontal coordinate indicates that the relative abundance of 12 genera changed significantly (*p* < 0.05), and the vertical coordinate indicates the time of fermentation, where darker color means stronger correlation, white means no correlation, red means positive correlation and blue means negative correlation.

Figure 2 shows the clustering heatmap of bacterial diversity at the genus level in fermented bamboo shoots. According to the variation analysis of the top 30 genera in the relative abundance of bacteria in bamboo shoots at three fermentation stages, it was found that the relative abundance of 12 genera changed significantly (*p* < 0.05). They were Streptococcus, *Leuconostoc*, *Acinetobacter*, *Lactococcus*, *Lactobacillus*, *Pseudomonas*, *Enterococcus*, *Escherichia-Shigella*, *Chishuiella*, *Clostridium-sensu-stricto-1*, *Chryseobacterium* and *Bacillus*. The relative abundance of opportunistic pathogens, such as *Streptococcus*, *Escherichia* and *Bacillus*, was higher in the early stage of fermentation but decreased significantly with prolonged fermentation time. In the late fermentation period of the bamboo shoots, LAB such as *Lactobacillus* and *Leuconostoc* were the dominant bacterial genera.

### 3.3. Bacterial Community Composition

**Figure 3:**
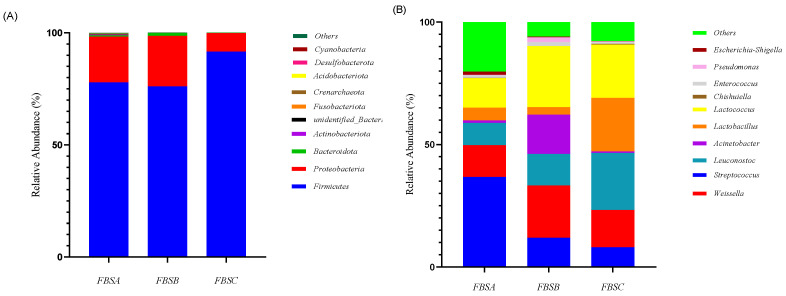
The relative abundance changes of bacteria at the phylum (**A**) and genus levels (**B**).

The relative abundance of bacteria in fermented bamboo shoots is shown in Figure 3. Figure 3A shows that Firmicutes (93.64%) was the dominant phylum and its relative abundance was highest at FBSC. The relative abundance of Proteobacteria increased to 30.34% at FBSB and then decreased significantly.

As shown in Figure 3B, there were mainly 10 bacterial genera with a relatively high relative abundance, accounting for 94.03% in total, which were the key bacterial genera in the fermented bamboo shoots. They were *Weissella*, *Streptococcus*, *Leuconostoc*, *Acinetobacter*, *Lactococcus*, *Lactobacillus*, *Pseudomonas*, *Enterococcus*, *Escherichia-Shigella* and *Chishuiella*. The top three genera in the relative abundance of FBSB were *Lactococcus* (24.23%), *Acinetobacter* (23.04%) and *Weissella* (18.81%). The top three genera in the relative abundance of FBSC were *Weissella* (25.11%), *Leuconostoc* (25.07%) and *Lactococcus* (19.43%). With the passage of fermentation time, the relative abundance of *Acinetobacter* and *Lactococcus decreased*, while the relative abundance of *Weissella*, *Leuconostoc* and *Lactobacillus* increased significantly (*p* < 0.05).

As shown in Figure 3, during the natural fermentation of bamboo shoots, the bacterial community structure and flavor substances changed significantly. In FBSB, the species of bacterial genera were the most abundant, which may be formed after mutual inhibition of bacterial growth, and it was in the middle of the process of fermentation dynamic change. As fermentation continued, the relative abundance of *Weissella* and *Leucanostoc* dominated, and bacterial diversity decreased. In the early stage of the bamboo shoots soaking in water, the content of dissolved oxygen decreased with the progress of fermentation, which inhibited the growth and reproduction of aerobic microorganisms, and facultative anaerobic lactic acid bacteria began to dominate [19]. The acid resistance and facultative anaerobicity of *Weissella* and *Lactobacillus* were stronger than those of *Lactococcus* [20], so the growth of *Lactococcus* was strongly inhibited and the relative abundance of *Lactobacillus* increased in FBSC. *Lactobacillus* and *Leucanostoc* are widely used as starter cultures in the food fermentation industry [21,22]. Xia et al. studied the diversity and dynamic changes of dominant bacteria during bamboo shoot curing and found that *L. lactis* was the dominant bacteria in the early stage of fermentation (7 days). Then, *Weissella* rapidly became dominant after 21 days and strongly inhibited the growth of L. lactis [23]. This was not consistent with the results of this study, indicating that the changes in microbial diversity in pickled bamboo shoots should be dependent on the special environment of high salt, high acid and low oxygen concentrations, which have a greater impact on the formation of nutrients and flavor.

### 3.4. Correlation Analysis between Bacterial Genera and VFCs

**Figure 4:**
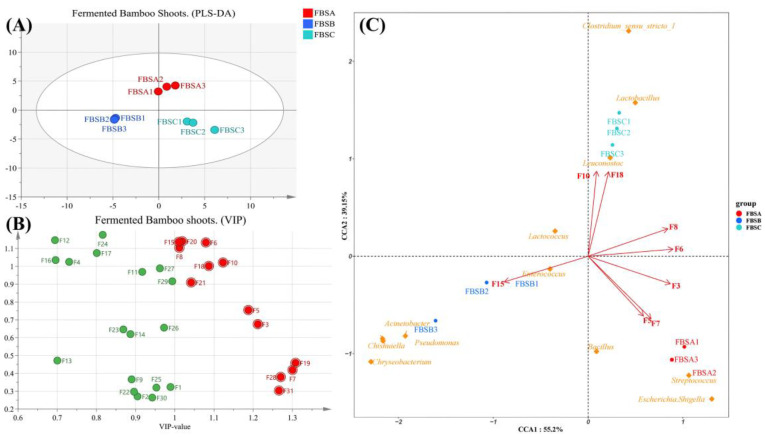
PLS-DA diagram of 3 group samples (**A**). VIP results of 31 volatiles (**B**). CCA analysis of samples, volatiles and bacterial genera (**C**). Note: Words in yellow indicate different bacterial genera and red arrows indicate VFCs. F1: Methyl salicylate. F2: 2-hydroxy-Benzoic acid phenylmethyl ester. F3: 3-methyl-1-Butanol. F4: 1-Octanol. F5: 1-Dodecanol. F6: Nonanal. F7: (E) -2-Octenal. F8: Decanal. F9: 6-methyl-5-Hepten-2-one. F10: acetic acid, 2-phenylethyl ester. F11: 2-Nonanol. F12: 1-Decanol. F13: Benzyl alcohol. F14: Benzaldehyde. F15: Benzene acetaldehyde. F16: Acetophenone. F17: 2-Methoxy-4-vinylphenol. F18: Benzoic acid, ethyl ester. F19: 1-Hexanol. F20: (E) -2-Nonenal. F21: 3-methyl-Benzaldehyde. F22: (E) -6,10-dimethyl-5,9-Undecadien-2-one. F23: Octanoic acid. F24: Nonanoic acid. F25: Acetic acid. F26: 2-hydroxy-Benzoic acid, pentyl ester. F27: 3-Octanol. F28: 1-Octen-3-ol. F29: Cycloheptanol. F30: p-Cresol. F31: Acetoin.

Figure 4A shows the distribution of VFCs in bamboo shoots at each fermentation stage obtained by partial least squares-discriminant analysis (PLS-DA). The three groups of fermented bamboo shoot samples were separated closely within groups and the spacing between the groups was large, which indicated that the reproducibility of each group was good. Variable importance in projection (VIP) was used to measure the impact of each metabolite accumulation difference on sample classification and its explanatory power in Figure 4B. VIP ≥ 1 is a screening criterion for common differential metabolites [24]. The red circle indicates the VFCs that changed significantly during fermentation (*p* < 0.01, VIP ≥ 1). They were 3-methyl-1-butanol, 1-dodecanol, nonanal, (E)-2-octenal, decanal, acetic acid, 2-phenylethyl ester, benzene acetaldehyde, benzoic acid, ethyl ester, 1-hexanol, (E)-2-nonenal, 3-methyl-benzaldehyde, 1-octen-3-ol and acetoin. The farther away from the origin, the greater its contribution to the flavor of bamboo shoots.

**Figure 5:**
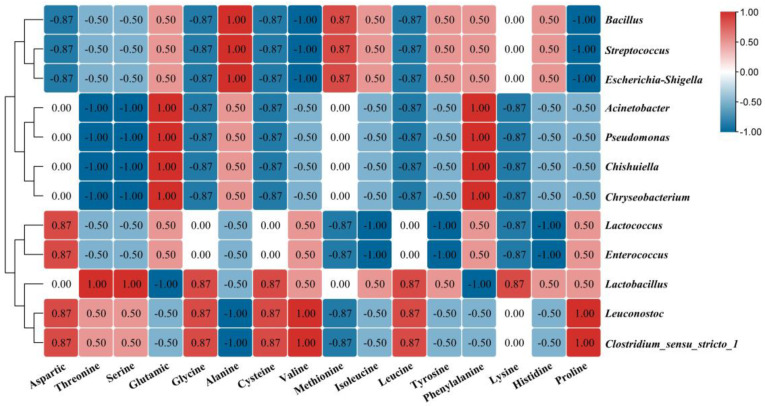
Heatmap between the bacterial genera and FAAs. Note: The color on the right and the number in the square represents ρ. The closer ρ was to 1 (red) indicated a positive correlation and the closer ρ was to −1 indicated a negative correlation (blue).

CCA between bacterial genera and VFCs was performed, as shown in Figure 4C, to further explore the relationship between abundant microorganisms and characteristic VFCs (VIP > 1). The longer the ray is, the greater the effect of the environmental factor. The correlation is positive when the angle is sharp, whereas an obtuse angle represents a negative correlation. As shown in Figure 5C, *Lactobacillus* and *Leuconostoc* showed the strongest positive correlation with acetic acid, 2-phenylethyl ester, benzoic acid, ethyl ester, nonanal and decanal. Lactococcus was positively correlated with benzene acetaldehyde, acetic acid, 2-phenylethyl ester, benzoic acid and ethyl ester. 3-methyl-1-butanol, 1-dodecanol and (E)-2-octenal showed a positive correlation with *Bacillus*, *Streptococcus* and *Escherichia_Shigella*.

Tyrosine can promote growth and is the starting point for plants to produce a variety of natural compounds, such as tocopherol and ubiquinone, which play an important role in plant survival [25,26]. In this study, tyrosine had the highest content and provided important growth components. Meanwhile, it is an aromatic amino acid and has a high TAV (4.95), which caused bamboo shoots to taste bitter. Aromatic amino acids can undergo transamino reactions to form phenylpyruvate, and the main products of degradation are hydroxyphenyllactic acid and hydroxyphenylacetic acid, followed by cinnamic acid and coumaric acid, which are the precursors of aromatic compounds [27,28]. The other way to produce VFCs, such as benzene acetaldehyde and benzaldehyde, is through the action of decarboxylase, which finally forms ester compounds such as ethyl phenylethyl ester [29]. Studies have shown that *L. lactis*, *L. delbrueckii* and *Streptococcus thermophilus* produce decarboxylase to decompose tyrosine to produce tyramine and aldehydes [30]. Phenylalanine is also an aromatic amino acid that can decarboxylate to produce VFCs containing benzene rings. Lactobacillus casei and Lactobacillus helveticus can carry out continuous transamino reactions and dehydrogenation reactions on phenylalanine in cheese and decompose it into benzoic acid, phenylacetic acid, phenyllactic acid and other VFCs [31]. In this study, Lactococcus was negatively correlated with tyrosine and positively correlated with benzene acetaldehyde, acetic acid, 2-phenylethyl ester, benzoic acid and ethyl ester. It can be speculated that the relative abundance of Lactococcus in fermented bamboo shoots increased significantly at FBSB, producing decarboxylase to decompose tyrosine and form VFCs, such as benzene acetaldehyde, which provided a grassy aroma to bamboo shoots.

### 3.5. The Changes in FAAs and the Correlation with Bacterial Genera

With the change in microbial flora, the corresponding flavor substances also changed. In this paper, this change was achieve through the change in amino acids to highlight that for different fermentation times of the amino acid, the changes were mainly through its TAV value to present.

In Table 1, a total of 16 kinds of FAAs were detected in the fermented bamboo shoots, among which tyrosine was the highest (450.57 ± 1.34 mg/100 g). During the natural fermentation of bamboo shoots for 36 h, most of the FAAs showed a significant decreasing trend. In FBSC, the contents of threonine, serine, valine, tyrosine and proline increased significantly (*p* < 0.05), and glutamic showed the opposite trend. Based on taste characteristics, histidine, isoleucine, leucine, lysine, methionine, phenylalanine and tyrosine were generally recognized as bitter amino acids [32]. The taste activity value (TAV) was calculated by using the ratio of each FAA to its sensory threshold in water [33]. The TAV of tyrosine (4.95) occupied first place, which had a slightly bitter taste sensation in fermented bamboo shoots and had an important impact on flavor.

Canned bamboo shoots in clear water have unique microecological characteristics in the fermentation proces, and special bacterial diversity changes have a vital impact on amino acid metabolism. Fermentation can produce essential amino acids with beneficial physiological functions. During 36 h natural fermentation of bamboo shoots, the contents of most FAAs decreased significantly, while the contents of threonine, serine, valine, tyrosine and proline increased significantly at 72 h (*p* < 0.05), This shows that the bitter substances were at their lowest level at 36 h and at the same time had a high freshness, providing a high nutritional value and taste experience to the palate of the bamboo shoot soaking juice.

Figure 5 displays the Spearman correlation analysis heatmap of bacterial genera and FAAs in fermented bamboo shoots. Cluster analysis showed that Bacillus, Streptococcus, and *Escherichia_Shigella* were negatively correlated with aspartic, glycine, cysteine, valine, leucine and proline but positively correlated with alanine and methionine. *Acinetobacter*, *Pseudomonas*, *Chishuiella* and *Chryseobacterium* were negatively correlated with threonine, serine, glycine, cysteine, leucine, and lysine but positively correlated with glutamic acid and phenylalanine. *Lactococcus* and Enterococcus were negatively correlated with methionine, isoleucine, tyrosine, lysine and histidine but positively correlated with aspartic. *Lactobacillus* was negatively correlated with glutamic and phenylalanine but positively correlated with threonine, serine, glycine, cysteine, valine, leucine and lysine. *Leuconostoc* and *Clostridium_sensu_stricto_1* were negatively correlated with alanine and methionine but positively correlated with alanine, glycine, cysteine, valine, leucine and proline.

Using Bacillus sp. fermented soy was a good way to provide a source of protein with species specificity. For example, *Bacillus licheniformis* can improve the contents of serine, threonine and glutamic acid in fermented soybean. Bacillus subtilis can increase the contents of alanine, aspartic acid, glycine and leucine [34]. In this study, *Bacillus* showed a negative correlation with aspartic acid, glycine, cysteine, valine, leucine and proline but a positive correlation with alanine and methionine, which was speculated to be caused by the different *Bacillus sp.* Cysteine is a tasteless amino acid but a flavor precursor and is positively correlated with *Lactobacillus.* Previous studies have shown that *Lactobacillus* paracasei has a cysteine synthesis pathway and genes similar to those of Escherichia coli which can promote the biosynthesis of cysteine in the fermentation process [35]. The formation of volatile thiols, with cysteine as the precursor, is an important contributor to the flavor of traditional fermented food and provides a sesame aroma to Baijiu [36]. Studies have shown that *Lactobacillus delbrueckii* subsp. Bulgaricus, which can generate C-S lyase, has broad substrate specificity for sulfur-containing amino acids and can decompose cysteine into thiols and improve food flavor [37].

### 3.6. Identification of L. lactis TJJ2

**Figure 6:**
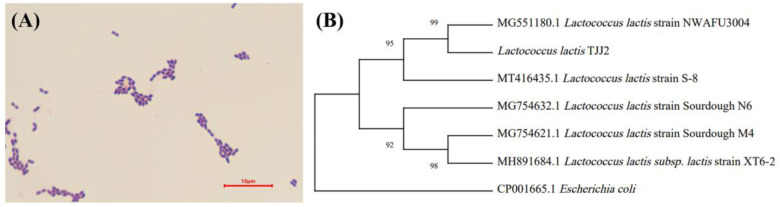
Morphological identification (**A**) and phylogenetic tree (**B**) of *L. lactis* TJJ2.

*Lactococcus* is positively correlated with aspartic but negatively correlated with methionine, isoleucine, tyrosine, lysine and histidine. We identified Lactococcus lactis as *L. lactis* TJJ2, which has more negatively correlated amino acids with *L. lactis* TJJ2, and we believe that this is related to the presentation of VFCS; it is the absence of this amino acid that leads to the alteration of the VFCs and we used *Lactococcus lactis* as a validation strain to supplement negatively correlated amino acids to the fermentation. The colonies were gray-white, opaque, with regular edges and smooth surfaces. Microscopic examination showed that a single strain was a Gram-positive spherical stain (Figure 6A). A comparison of the developmental trees established by the BLAST procedure showed that the sequence similarity between this strain and *L. lactis*, which was isolated from fermented foods, was 100% and confirmed that the screened strain was *L. lactis* (Figure 6B). All *L. lactis* sequences were sourced from fermented foods. According to Table 2, it could be concluded that the target strain accords with the characteristics of *L. lactis*.

### 3.7. Decarboxylase Validation Analysis

**Figure 7:**
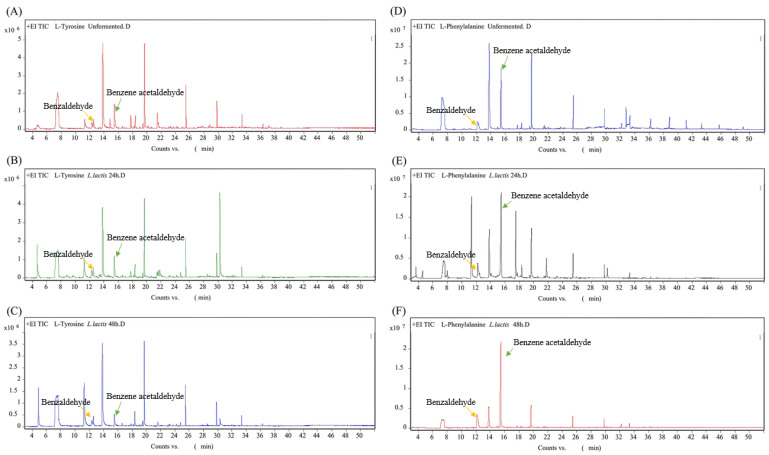
Ion flow diagram of VFCs fermented by *L. lactis.* L-tyrosine decarboxylase liquid medium fermented by *L. lactis* (**A**–**C**); L-Phenylalanine decarboxylase liquid medium fermented by *L. lactis* (**D**–**F**).

Figure 7A–C shows the ion flow diagram of VFCs in the L-Tyrosine decarboxylase liquid medium inoculated with *L. lactis* from bamboo shoots. The peak time of benzaldehyde was 12.27 min and the peak area of benzaldehyde was 2.71 × 106 without fermentation. After 24 h of fermentation, the peak area increased significantly to 6.16 × 106 and then decreased to 4.07 × 106 at 48 h. Other alcohols, aldehydes and esters containing benzene rings were not detected.

Figure 7D–F shows the ion flow diagram of VFCs in the L-Phenylalanine decarboxylase liquid medium inoculated with *L. lactis* from bamboo shoots. The peak time of benzene acetaldehyde was 15.53 min. The peak area in the unfermented decarboxylase liquid medium was 1.15 × 108. After 24 h of fermentation by *L. lactis*, the peak area increased to 1.46 × 108 and then decreased after fermentation for 48 h.

**Figure 8:**
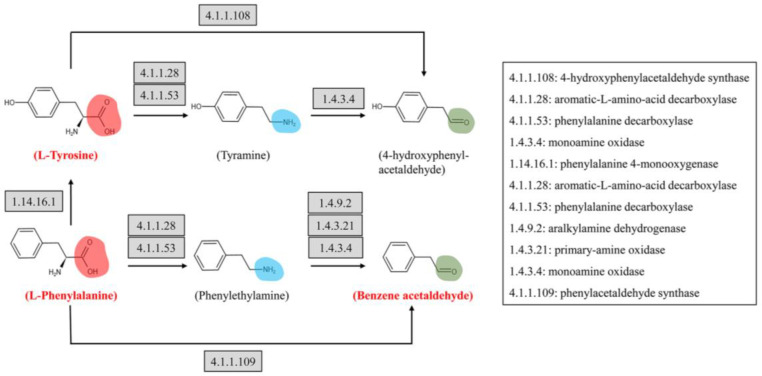
Decarboxylase test pathway diagram.

The verification test of tyrosine and phenylalanine decarboxylase showed that the fermentation process of *L. lactis* from bamboo shoots could produce decarboxylase and degrade tyrosine and phenylalanine to increase the benzene acetaldehyde and benzaldehyde in the fermentation broth (Figure 8). However, with the prolongation of fermentation time, benzene acetaldehyde and benzaldehyde could be oxidized to acids and their contents decreased. The benzene acetaldehyde and benzaldehyde contained in the uninoculated fermentation broth may be produced by tyrosine decomposition during high-temperature sterilization [38,39].

*Lactobacillus* was negatively correlated with phenylalanine but positively correlated with acetic acid, 2-phenylethyl ester and ethyl benzoate. It can be speculated that Lactobacillus produced transaminase during the fermentation of bamboo shoots, removed the amino radical in phenylalanine, and continuously dehydrogenated to produce substances such as benzaldehyde and benzene acetaldehyde. Aldehydes are unstable and can be oxidized to acids, and then acetic acid, a 2-phenylethyl ester, is produced by esterification with alcohols. The relative abundance of *Lactobacillus* in bamboo shoots increased at FBSC, and acetic acid and 2-phenylethyl ester were produced to provide flower flavor to bamboo shoots. However, the subsequent verification test results showed that Lactobacillus fermentum and L. brevis could not produce VFCs containing benzene rings in the phenylalanine decarboxylase liquid medium. It is speculated that there are other strains of Lactobacillus from bamboo shoots to carry out this link, or that the conclusion of correlation analysis between microbial diversity and amino acid metabolism cannot be obtained by fermentation alone, indicating that the limitations of correlation statistical analysis can only play a directional role for follow-up research; therefore, a verification test is necessary. On the other hand, L. fermentum and L. lactis left the original microbial diversity environment and no other microorganisms worked together to obtain statistical analysis conclusions.

## 4. Conclusions

This study presented flavor changes, bacterial community succession and their relationship during fermentation of bamboo shoots in clear water on an industrial scale. Then, we verified and explored the pathway of *L. lactis* from bamboo shoots using tyrosine to produce benzaldehyde and benzene acetaldehyde in a decarboxylase liquid medium. According to the results of this study, the production process of fermented bamboo shoots can be optimized, such as to strengthen the grass fragrance and relieve flavor fading by inoculating *L. lactis*. The changes in other FAAs and their related VFCs caused by microbes are worthy of further verification in subsequent studies and will also be conducive to the improvement of fermented bamboo shoot products.

## Figures and Tables

**Table 1:** Changes in FAA contents and TAV during fermentation.

Taste	Nutrients	Sensory Threshold mg/100 mL	FBSA		FBSB		FBSC	
			Contents mg/100 g	TAV	Contents mg/100 g	TAV	Contents mg/100 g	TAV
Umami	Aspartic	100	0.78 ± 0.02 ^b^	0.01	2.10 ± 0.10 ^a^	0.02	2.20 ± 0.10 ^a^	0.02
	Glutamic	30	11.13 ± 1.01 ^b^	0.37	54.00 ± 1.00 ^a^	1.8	7.41 ± 0.41 ^c^	0.25
Sweet	Threonine	260	10.00 ± 0.20 ^b^	0.04	7.03 ± 0.25 ^c^	0.03	16.00 ± 0.50 ^a^	0.06
	Serine	150	12.13 ± 1.01 ^b^	0.08	11.00 ± 0.50 ^b^	0.07	32.07 ± 1.01 ^a^	0.21
	Glycine	130	1.23 ± 0.65 ^a^	0.01	1.17 ± 0.25 ^a^	0.01	1.60 ± 0.36 ^a^	0.01
	Alanine	60	14.00 ± 0.10 ^a^	0.23	12.00 ± 1.00 ^b^	0.2	10.07 ± 0.90 ^c^	0.17
	Proline	300	ND		5.87 ± 0.55 ^b^	0.02	16.03 ± 0.85 ^a^	0.05
Bitter	Histidine	20	6.60 ± 0.66 ^a^	0.33	4.50 ± 0.95 ^b^	0.23	5.80 ± 0.70 ^ab^	0.29
	Isoleucine	90	8.73 ± 0.15 ^a^	0.01	5.10 ± 0.10 ^c^	0.06	7.17 ± 0.70 ^b^	0.08
	Leucine	190	4.40 ± 0.90 ^a^	0.02	4.03 ± 0.95 ^a^	0.02	5.57 ± 1.01 ^a^	0.03
	Lysine	50	3.00 ± 0.20 ^a^	0.06	2.23 ± 0.25 ^a^	0.04	3.00 ± 0.60 ^a^	0.06
	Methionine	30	2.13 ± 0.15 ^a^	0.07	ND		ND	
	Phenylalanine	90	4.26 ± 0.55 ^b^	0.05	6.14 ± 0.59 ^a^	0.07	ND	
	Tyrosine	91	450.57 ± 1.34 ^a^	4.95	270.67 ± 2.08 ^c^	2.97	360.23 ± 2.16 ^b^	3.96
Tasteless	Cysteine	-	0.64 ± 0.11 ^a^	-	1.22 ± 0.09 ^a^	-	1.50 ± 0.21 ^a^	-
	Valine	-	5.57 ± 0.35 ^b^	-	6.67 ± 0.80 ^b^	-	12.03 ± 1.00 ^a^	-

Note: ND represents not detected; ^a,b,c^ represent significant differences. (*p* < 0.05).

**Table 2:** Biochemical identification table of *L. lactis* TJJ2.

Carbon Source	Growth	Carbon Source	Growth	Carbon Source	Growth	Carbon Source	Growth
CK	-	D-Galactose	+	D-Melibiose	-	L-Arabinitol	-
Glycerol	-	D-Glucose	+	Sucrose	+	Salicin	+
Erythritol	-	D-Fructose	+	D-Trehalose	-	D-Cellobiose	+
D-Arabinose	-	D-Mannose	+	Inulin	-	D-Arabinitol	-
L-Arabinose	-	L-Sorbose	-	D-Melezitose	-	L-Rhamnose	-
D-Ribose	+	Esculin ferric citrate	+	D- Gentiobiose	+	Potassium Gluconate	-
D-Xylose	-	Dulcitol	-	Starch	+	L-Fucose	-
L-Xylose	-	Inositol	-	Glycogen	-	Arbutin	-
D-Ribose	-	Mannitol	-	Xylitol	-	D-Maltose	+
D-Lactose	+	Sorbitol	-	D-Raffinose	-	D-Fucose	-
α-Methyl-D-mannoside	-	D-Turanose	-	Potassium 5-ketogluconate	-	Amygdalin	+
α-Methyl-D-glucoside	-	N-Acetylglucosamine	+	β-Methyl-D-xyloside	-	Potassium 2-ketogluconate	-
D-Lyxose	-	D-Tagatose	-				

Note: + means positive, - means negative.

## Data Availability

The data presented in this study are available on request from the corresponding author.

## References

[1] Behera S.S., El Sheikha A.F., Hammami R., Kumar A. (2020). Traditionally fermented pickles: How the microbial diversity associated with their nutritional and health benefits?. J. Funct. Foods.

[2] Du R.P., Song G., Zhao D., Sun J., Ping W.X., Ge J.P. (2018). *Lactobacillus casei* starter culture improves vitamin content, increases acidity and decreases nitrite concentration during sauerkraut fermentation. Int. J. Food Sci. Technol..

[3] Darmayanti L.P.T., Duwipayana A.A., Putra I.N.K., Antara N.S. (2014). Preliminary Study of Fermented Pickle of Tabah Bamboo Shoot (*Gigantochloa nigrociliata* (Buese) Kurz). Int. J. Biol. Vet. Agric. Food Eng..

[4] Sarangthem K., Singh T. (2013). Fermentation decreases the anti-nutritional content in bamboo shoots. Int. J. Curr. Microbiol. Appl. Sci..

[5] Sonar N.R., Vijayendra S.V.N., Prakash M., Saikia M., Tamang J.P., Halami P.M. (2015). Nutritional and functional profile of traditional fermented bamboo shoot based products from Arunachal Pradesh and Manipur states of India. Int. Food Res. J..

[6] Devi S.P., Singh H.T. (1986). Studies on the chemical and nutritional changes of bamboo shoots during fermentation. J. Food Sci. Technol..

[7] Agrahar-Murugkar D., Subbulakshmi G. (2006). Preparation techniques and nutritive value of fermented foods from the Khasi tribes of Meghalaya. Ecol. Food Nutr..

[8] Zang J., Yu D., Zhang P., Xu Y., Xia W. (2022). The key enzymes and flavor precursors involved in formation of characteristic flavor compounds of low-salt fermented common carp (*Cyprinus carpio* L.). LWT.

[9] Procopio S., Brunner M. (2014). Differential transcribed yeast genes involved in flavour formation and its associated amino acid metabolism during brewery fermentation. Eur. Food Res. Technol..

[10] Badwaik L.S., Borah P.K., Borah K., Das A.J., Deka S.C., Sharma H.K. (2014). Influence of Fermentation on Nutritional Compositions, Antioxidant Activity, Total Phenolic and Microbial Load of Bamboo Shoot. Food Sci. Technol. Res..

[11] Tamang B., Tamang J.P. (2009). Lactic Acid Bacteria Isolated from Indigenous Fermented Bamboo Products of Arunachal Pradesh in India and Their Functionality. Food Biotechnol..

[12] Hata T., Alemu M., Kobayashi M., Suzuki C., Nitisinprasert S., Ohmomo S. (2009). Characterization of a Bacteriocin Produced by Enterococcus faecalis N1-33 and Its Application as a Food Preservative. J. Food Prot..

[13] Tang J., Zhang Z., Zheng S., Gao N., Li Z., Li K. (2021). Changes of Main Nutrient Components and Volatile Flavor Substances in Processing of Canned Bamboo Shoots. Fermentation.

[14] Minas K., McEwan N.R., Newbold C.J., Scott K.P. (2011). Optimization of a high-throughput CTAB-based protocol for the extraction of qPCR-grade DNA from rumen fluid, plant and bacterial pure cultures. FEMS Microbiol. Lett..

[15] Chen C., Zhang J., Lu M., Qin C., Chen Y., Yang L., Huang Q., Wang J., Shen Z., Shen Q. (2016). Microbial communities of an arable soil treated for 8 years with organic and inorganic fertilizers. Biol. Fertil. Soils.

[16] Schloss P.D., Westcott S.L., Ryabin T., Hall J.R., Hartmann M., Hollister E.B., Lesniewski R.A., Oakley B.B., Parks D.H., Robinson C.J. (2009). Introducing mothur: Open-source, platform-independent, community-supported software for describing and comparing microbial communities. Appl. Environ. Microbiol..

[17] Chen H.C., Yi Z., Hannah R. (2020). TBtools: An Integrative Toolkit Developed for Interactive Analyses of Big Biological Data. Mol. Plant.

[18] Guan-Yu F., Li-Juan C., Xiao-Zhong Z., Yu-Jian J. (2021). Deciphering the succession patterns of bacterial community and their correlations with environmental factors and flavor compounds during the fermentation of Zhejiang rosy vinegar. J. Int. J. Food Microbiol..

[19] Chi H., Lu W.W., Liu G.Q., Qin Y.Y. (2020). Physiochemical property changes and mineral element migration behavior of bamboo shoots during traditional fermentation process. J. Food Process. Preserv..

[20] Park J.H., Ahn H.J., Kim S.G., Chung C.H. (2013). Dextran-like exopolysaccharide-producing *Leuconostoc* and *Weissella* from kimchi and its ingredients. Food Sci. Biotechnol..

[21] Lu H., Huang C., Yu K., Liu Z. (2022). Effects of Mixed Inoculation of *Leuconostoc Citreum* and *Lactobacillus Plantarum* on Suansun (Sour Bamboo Shoot) Fermentation. Food Biosci..

[22] Montemurro M., Celano G., Angelis M., Gobbetti M., Pontonio E.J.F.M. (2020). Selection of non-Lactobacillus strains to be used as starters for sourdough fermentation. Food Microbiol..

[23] Xia X.J., Ran C.X., Ye X.J., Li G.N., Kan J.Q., Zheng J. (2017). Monitoring of the bacterial communities of bamboo shoots (*Dendrocalamus latiflorus*) during pickling process. Int. J. Food Sci. Technol..

[24] Zhong A., Chen W., Duan Y., Li K., Tang X., Tian X., Wu Z., Li Z., Wang Y., Wang C. (2021). The potential correlation between microbial communities and flavors in traditional fermented sour meat. LWT.

[25] Singhal P., Bal L.M., Satya S., Sudhakar P., Naik S.N. (2013). Bamboo Shoots: A Novel Source of Nutrition and Medicine. Crit. Rev. Food Sci. Nutr..

[26] Xu J.-J., Fang X., Li C.-Y., Yang L., Chen X.-Y. (2019). General and specialized tyrosine metabolism pathways in plants. Abiotech.

[27] Fernandez M., Zuniga M. (2006). Amino acid catabolic pathways of lactic acid bacteria. Crit. Rev. Microbiol..

[28] Jendresen C.B., Stahlhut S.G., Li M., Gaspar P., Siedler S., Forster J., Maury J., Borodina I., Nielsen A.T. (2015). Highly Active and Specific Tyrosine Ammonia-Lyases from Diverse Origins Enable Enhanced Production of Aromatic Compounds in Bacteria and *Saccharomyces cerevisiae*. Appl. Environ. Microbiol..

[29] Tieman D., Taylor M., Schauer N., Fernie A.R., Hanson A.D., Klee H.J. (2006). Tomato aromatic amino acid decarboxylases participate in synthesis of the flavor volatiles 2-phenylethanol and 2-phenylacetaldehyde. Proc. Natl. Acad. Sci. USA.

[30] Buňková L., Buňka F., Hlobilová M., Vaňátková Z., Nováková D., Dráb V. (2009). Tyramine production of technological important strains of Lactobacillus, Lactococcus and Streptococcus. Eur. Food Res. Technol..

[31] Gummalla S., Broadbent J.R. (2001). Tyrosine and Phenylalanine Catabolism by Lactobacillus Cheese Flavor Adjuncts. J. Dairy Sci..

[32] Li K., Tang J.J., Zhang Z.X., Wu Z.Q., Zhong A.A., Li Z.J., Wang Y.L. (2022). Correlation between flavor compounds and microorganisms of Chaling natural fermented red sufu. LWT Food Sci. Technol..

[33] Zhou Z.L., Liu S.P., Kong X.W., Ji Z.W., Han X., Wu J.F., Mao J. (2017). Elucidation of the aroma compositions of Zhenjiang aromatic vinegar using comprehensive two dimensional gas chromatography coupled to time-of-flight mass spectrometry and gas chromatography-olfactometry. J. Chromatogr. A.

[34] Jang M., Jeong D.W., Heo G., Kong H., Kim C.T., Lee J.H. (2021). Genetic Background Behind the Amino Acid Profiles of Fermented Soybeans Produced by Four *Bacillus* spp.. J. Microbiol. Biotechnol..

[35] Wuthrich D., Wenzel C., Bavan T., Bruggmann R., Berthoud H., Irmler S. (2018). Transcriptional Regulation of Cysteine and Methionine Metabolism in *Lactobacillus paracasei* FAM18149. Front. Microbiol..

[36] Shen T., Liu J., Wu Q., Xu Y. (2020). Increasing 2-furfurylthiol content in Chinese sesame-flavored Baijiu via inoculating the producer of precursor l-cysteine in Baijiu fermentation. Food Res. Int..

[37] Allegrini A., Astegno A., La Verde V., Dominici P. (2017). Characterization of C-S lyase from *Lactobacillus delbrueckii* subsp. bulgaricus ATCC BAA-365 and its potential role in food flavour applications. J. Biochem..

[38] Hidalgo F.J., Alcón E., Zamora R. (2013). Cysteine- and serine-thermal degradation products promote the formation of Strecker aldehydes in amino acid reaction mixtures. Food Res. Int..

[39] Tang H., Li P., Chen L., Ma J.-K., Guo H.-H., Huang X.-C., Zhong R.-M., Jing S.-Q., Jiang L.-W. (2022). The formation mechanisms of key flavor substances in stinky tofu brine based on metabolism of aromatic amino acids. Food Chem..

